# Steady State of Motion of Two Particles in Poiseuille Flow of Power-Law Fluid

**DOI:** 10.3390/polym14122368

**Published:** 2022-06-11

**Authors:** Dongmei Chen, Jianzhong Lin

**Affiliations:** 1 State Key Laboratory of Fluid Power Transmission and Control, Zhejiang University, Hangzhou 310027, China; chendm@zju.edu.cn; 2 Laboratory of Impact and Safety Engineering of Ministry of Education, Ningbo University, Ningbo 315201, China

**Keywords:** particle motion, steady state, Poiseuille flow, power-law fluid, numerical simulation

## Abstract

The steady state of motion of two particles in Poiseuille flow of power-law fluid is numerically studied using the lattice Boltzmann method in the range of Reynolds number 20 ≤ *Re* ≤ 60, diameter ratio of two particles 0.125 ≤ *β* ≤ 2.4, and power-law index of the fluid 0.4 ≤ *n* ≤ 1.2. Some results are validated by comparing with other available results. The effects of *Re*, *β,* and *n* on the steady state of motion of two particles are discussed. The results show that, for two particles of the same diameter, the particle spacing *l* in the steady state is independent of *n*. In shear-thinning fluid, *l* increases rapidly at first and then slowly, finally approaching a constant for different *Re*. In shear-thickening fluid, although *l* tends to be stable in the end, the values of *l* after stabilization are different. For two particles of different sizes, *l* does not always reach a stable state, and whether it reaches a stable state depends on *n*. When the small particle is downstream, *l* increases rapidly at first and then slowly in shear-thickening fluid, but increases rapidly at first and then decreases slowly, finally approaching a constant in a shear-thinning fluid. In shear-thinning fluid, the larger *n* is, the smaller *l* is. In shear-thickening fluid, *β* has no effect on *l* in steady-state. When the large particle is downstream, *l* increases rapidly at first and then slowly in shear-thinning fluid but increases rapidly at first and then decreases in a shear-thickening fluid. The effect of *n* on *l* in the steady state is obvious. In shear-thinning fluid, *l* increases rapidly at first and then slowly, the larger *Re* is, the smaller *l* is. In shear- thickening fluid, *l* will reach a stable state.

## 1. Introduction

Just like the application of electronic robust control in the field of computing and communication, the precise and programmable control of the equilibrium position of microdroplets or particles in a channel or pipe has been applied to the field of biomedicine, materials synthesis, and so on [1]. Mastering the migration characteristics of randomly distributed particles towards equilibrium positions has gained increasing attention, based on which technology can be developed to count, focus and separate particles with high efficiency. With the rapid development of microfluidic chips, the basic research of microscale flow has gradually formed a scale. Microfluidic chips transport fluids at the microscale. As a platform, through the manipulation of the flow, various functions such as chemical analysis, drug screening, and cell culture can be realized. This kind of microscale system involves chemical and biological flow, so it is necessary to carry out research on the properties of microscale flow of non-Newtonian fluid or biological fluid [2,3].

Due to its fundamental and practical significance, there have been a large number of theoretical, experimental, and numerical studies on the migration of rigid particles in a confined flow. About the migration of particles in a Newtonian fluid, we can get some intuitions or results from available experimental and numerical results [1,4,5,6,7], but in non-Newtonian fluids, the results could be completely different [8,9,10,11,12,13]. Non-Newtonian fluids have significantly different rheological attributes than Newtonian fluids in their viscoelasticity and their apparent viscosity varies with shear rate. When particles migrate in non-Newtonian fluids, the migration properties would change significantly. However, non-Newtonian fluids are very common in engineering applications and daily life. As a special non-Newtonian fluid, power-law fluids exhibit shear-thinning or shear-thickening properties. For example, blood is a kind of shear-thinning fluid, it is critical to study the flow of blood cells and the migration of viruses or drugs in blood. Chrit et al. [14] studied the migration of particles in a two-dimensional Poiseuille flow of power-law fluid using the lattice Boltzmann method, and it turned out that the velocity profile of power-law fluid is different from that of a Newtonian fluid, so the force exerted on the particles by the fluid is different because of different velocity gradients around the particles. As a result, the lateral equilibrium position of the particles is changed, i.e., the equilibrium position of the particles in the shear-thinning fluids is closer to the wall than that in the shear-thickening fluids.

Most of the previous studies focused on the flows with low Reynolds numbers, and the inertia can be ignored in such flows [8,15,16]. Although the characteristic scale is relatively small in microchannel flow, the fluid velocity is sometimes large. In this case, the Reynolds number is not small, so the inertia cannot be ignored. Therefore, the effect of the inertial force should be considered in the design of the microfluidic chip, thus the fluid inertia can be used to control the equilibrium position of particles in the microchannel [3]. Particle dynamics are complicated because of the instability and aggregation of particle interactions caused by fluid inertia. The inertial effect was first discovered by Segre’ and Silberberg [17], i.e., the particles flowing in the circular tube will spontaneously aggregate to the position of 0.6 times the pipe diameter, which is known as the “tubular pinch effect”. Behind this particle, the focus is the combined effect of fluid shear, the constraint of the tube wall, and particle rotation. More interestingly, the particles not only migrate to the equilibrium position of the cross-section by inertia but also self-assemble into uniformly spaced particle trains in the flow direction due to the interactions between particles. Matas et al. [18] found experimentally that a stable uniformly spaced particle train is formed during the inertial migration of particles in both straight and curved pipes, which was confirmed by Di Carlo et al. [19]. Humphry et al. [5] stated that the ability of particles to form trains is related to the particle number in the channel flow. Through a combination of experiments and numerical simulations, they captured the evolution of the equilibrium position of the particles in the rectangular channel and provided some guidance for the formation of particle trains. In recent years, efforts have been made to understand more about this organized state [4,5,7,8,10,18,19,20,21,22,23,24,25,26,27].

To better understand the interactions between particles, and between particles and fluid, as well as the formation mechanism of a stable particle train, we first studied the motion of two particles in the Poiseuille flow at a finite Reynolds number and provided a preliminary understanding of the mechanism through the interaction of two particles. It is possible to obtain predictions of the attractive or repulsive interaction of particles. For two particles, the results are different when the particles are initially on or not on the same horizontal line. Schaaf et al. [28] studied the stability of the relative position of two particles under different initial conditions and found that the relative position of two particles is stable and anti-interference when the particles are initially not on the same horizontal line, while they could only be stable on one side but not anti-interference when the particles are initially on the same horizontal line. Hood and Roper [29] indicated that both cases are stable when the particles are initially on or not on the same horizontal line, with viscosity acting as the dominant (first-order) analogous to spring motion, and inertia acting as a perturbation (second-order) analogous to friction damping. When the particles are initially on the same horizontal line, the particle motion is much like a damped harmonic oscillation, or a spring motion with frictional resistance. The formation of the stable relative position of two particles is a result of the minimization of the kinetic energy of the fluid. Two and three particles can form stable relative positions and give the acceleration curves of each particle. In the process of forming a stable relative position, the repulsion is synchronous, and the attraction is asynchronous, but eventually, a stable relative position is formed. In a previous study [30], we studied the migration of particles of different sizes in a Newtonian fluid. Hu et al. [31] found that the particles bear great resistance when moving in shear-thickening fluids, the inertial migration of particles to form particle train is slower than that in shear-thinning fluids.

It can be seen from the above studies that there is still a lack of research on the formation of the stable relative position of two particles with different sizes in power-law fluid, and the effects of fluid characteristics, particle size, and flow conditions on the stable relative position of two particles have not been reported. Exploring the steady state of particles migrating in the flow has practical significance for particle detection, separation, focusing, and counting in an application. Therefore, the aim of this study is to assess the steady state of motion of two particles in the Poiseuille flow of power-law fluid and explore the effects of the power-law index of the fluid, Reynolds number, diameter ratio of two particles on the steady state of motion of two particles. We focus on the Poiseuille flow because it is very common in practical applications [32].

## 2. Numerical Model

### 2.1. Lattice Boltzmann Method

The lattice Boltzmann method (LBM) is used to numerically simulate the neutral suspended particles in the Poiseuille flow of power-law fluid. The LBM is a new type of hydrodynamic numerical method based on the molecular dynamics theory at the mesoscopic scale and has proven to be an effective method in the simulation of two-phase flow [33]. Its main variables are the density distribution functions in several discrete velocity directions. The viscous incompressible flow can be expressed by a single relaxed lattice Boltzmann equation with an external force term as:

(1) $$f_{i}\left(x+\Delta te_{i},t+\Delta t\right)=f_{i}\left(x,t\right)+\frac{1}{\tau }\left[f_{i}^{eq}\left(x,t\right)-f_{i}\left(x,t\right)\right]+\Delta t\cdot F_{p}$$

where *f_i_*(***x***,*t*) is the distribution function for the microscopic velocity ***e****_i_* in the*i*th direction; *τ* is the dimensionless relaxation time *τ* = *τ*_0_/∆*t*(*τ*_0_ is collision time); *f_i_^eq^*(***x***,*t*)is the equilibrium distribution function; Δ*t* is the unit time step; *F*_p_ is the external force term.

The equilibrium distribution function following the D3Q19 model proposed by Qian et al. [34] is:

(2) $$f_{i}^{eq}=\rho w_{i}\left[1+\frac{e_{i}\cdot u}{c_{s}^{2}}+\frac{{\left(e_{i}\cdot u\right)}^{2}}{2c_{s}^{4}}-\frac{u^{2}}{2c_{s}^{2}}\right],c_{s}=\frac{1}{\sqrt{3}},w_{0}=\frac{1}{3},w_{1\sim 6}=\frac{1}{18},w_{7\sim 18}=\frac{1}{36}$$

where *c_s_* is the speed of sound; *w_i_* is the weight factor; *ρ* and *u* represent the fluid density and velocity, respectively.

The speed configuration of the D3Q19 model is as follows:

(3) $$E=\left[\begin{array}{ccccccccccccccccccc} 0 & 1 & -1 & 0 & 0 & 0 & 0 & 1 & -1 & 1 & -1 & 1 & -1 & 1 & -1 & 0 & 0 & 0 & 0\\ 0 & 0 & 0 & 1 & -1 & 0 & 0 & 1 & -1 & -1 & 1 & 0 & 0 & 0 & 0 & 1 & -1 & 1 & -1\\ 0 & 0 & 0 & 0 & 0 & 1 & -1 & 0 & 0 & 0 & 0 & 1 & -1 & -1 & 1 & 1 & -1 & -1 & 1 \end{array}\right]$$

The external force term with good stability proposed by He et al. [35] is:

(4) $$F_{p}=\left(1-\frac{1}{2\tau }\right)\frac{\left(e_{i}-u\right)\cdot F_{b}}{c_{s}^{2}}f_{i}^{eq}\left(x,t\right)$$

where ***F_b_*** is the body force. The fluid density (total number of particles) and velocity satisfy:

(5) $$\rho =\sum f_{i},\;u=\frac{1}{\rho }\sum f_{i}e_{i}+\frac{\Delta t}{2\rho }F_{b}$$

The macroscopic pressure is given directly by the equation of state by *p* = *ρc_s_^2^*.

By performing a Chapman–Enskog expansion, the macroscopic mass and momentum equations in the low Mach number limit can be recovered and have second-order accuracy in both time and space:

(6) ∇⋅u=0

(7) $$\rho \frac{Du}{Dt}=-\nabla p+\rho f+\nabla \cdot \tau$$

where ***u*** is the velocity; *ρ* is the fluid density; *p* is the pressure; *f* is the body force; *τ* is the shear stress and given by $\tau =\mu \dot{\gamma }$ with *μ* the dynamic viscosity and $\dot{\gamma }$ the rate of shearing tensor:

(8) $$\dot{\gamma }=\frac{1}{2}\left[\left(\nabla u\right)+{\left(\nabla u\right)}^{T}\right]$$

the power-law fluid model is expressed as:

(9) $$\tau =m{\left|\dot{\gamma }\right|}^{n-1}\dot{\gamma }$$

The effective viscosity is related to the shearing rate of the fluid by:

(10) $$\mu =m{\left|\dot{\gamma }\right|}^{n-1}$$

where *m* is the flow consistency coefficient; *n* is the power-law index, *n* = 1 corresponds to Newtonian fluid, and *n* < 1 and *n* > 1 correspond to shear-thinning and shear-thickening fluids, respectively; $\left|\dot{\gamma }\right|$ is the local shear rate and can be obtained from the strain rate tensor:

(11) $$\left|\dot{\gamma }\right|=\sqrt{2D:D}=\sqrt{2{\left(\frac{\partial u}{\partial x}\right)}^{2}+2{\left(\frac{\partial v}{\partial y}\right)}^{2}+{\left(\frac{\partial u}{\partial x}+\frac{\partial v}{\partial y}\right)}^{2}}$$

where D is the velocity gradient tensor $\left|\dot{\gamma }\right|$ can be computed from a fourth-order finite-difference approximation to the local derivatives of the velocity:

(12) $$\frac{\partial u}{\partial x}=\frac{2}{3\Delta x}\left(u_{i+1,j,k}-u_{i-1,j,k}\right)+\frac{1}{12\Delta x}\left(u_{i+2,j,k}-u_{i-2,j,k}\right)+O\left(\Delta x^{4}\right)$$

(13) $$\frac{\partial v}{\partial y}=\frac{2}{3\Delta x}\left(u_{i,j+1,k}-u_{i,j-1,k}\right)+\frac{1}{12\Delta x}\left(u_{i,j+2,k}-u_{i,j-2,k}\right)+O\left(\Delta x^{4}\right)$$

The instantaneous local relaxation time for each fluid lattice point can be obtained by *ν* =(2*τ_f_*−1)*c*^2^Δ*t*/6, where *τ_f_* is the instantaneous local relaxation time for each fluid lattice point and *c* is the ratio of grid step to time step.

For shear-thinning fluids, at the zero-shear rate fluid lattice point, its apparent viscosity tends to infinity and diverges. For shear-thickening fluids, at the zero-shear rate fluid lattice point, its apparent viscosity will be equal to zero. Both of these cases can lead to unstable or low computational accuracy of the LBM. Therefore, the upper and lower limits of apparent viscosity can be set, respectively [36]:

(14) $${\left[\frac{\mu \left(x\right)}{\rho }\right]}_{\min}=0.001;{\left[\frac{\mu \left(x\right)}{\rho }\right]}_{\max}=0.1$$

### 2.2. Fluid-Particle Coupling and Boundary Treatment

When the LBE is applied, the boundary conditions of the distribution function need to be given. The velocity boundary can be divided into the straight boundary and the curved boundary. At the exit and entrance, the periodic boundary conditions are used:

(15) $$f_{i}\left(x,t+\Delta t\right)=f_{i}^{*}[\left(x-e_{i}\Delta t+x_{T}\right)\%x_{T},t]$$

where * represents the step after collision; *x_T_* is the vector composed of periodic lengths in each coordinate direction; % is the remainder operation.

The periodic scheme assumes that the fluid and particles leave the flow from one boundary and re-enter the flow from the other side of the flow at the next time step, obtaining an infinite domain in the flow direction, which can strictly guarantee the conservation of mass and momentum at the boundary.

A no-slip boundary condition is applied in this paper. The reason is that although microchannels are studied here, the Kn number (the ratio of the average free path of molecules to the characteristic length of the flow) is far less than 0.001. For the no-slip condition on the walls, the standard bounce format is used:

(16) $$f_{i}\left(x,t+\Delta t\right)=f_{i’}^{*}\left(x,t\right)$$

which is based on the reflection principle, i.e., using the distribution function of the boundary nodes after the collision step to obtain the unknown partial function.

For the numerical simulation method that adopts the full analysis of particles, the accurate analysis of the boundary conditions of the particles plays a crucial role in the calculation accuracy. Ladd [37] proposed a half-way bounce scheme thatis suitable for moving boundaries:

(17) $$f_{i’}\left(x,t+\Delta t\right)=f_{i}^{*}\left(x,t_{+}\right)-2B_{i}\left(e_{i}\cdot u_{b}\right)$$

where *i*′ and *i* are the reflected and incident directions, respectively; *t*_+_ is the post-collision time; *B_i_* = 3*ρω_i_*/*c*^2^; *u_b_* = *u*_0_ + Ω × *x_b_*, *u*_0_ is the translational velocity of the mass center of the particle, and Ω is the angular velocity; *x_b_* = *x* + Δ*te_i_*/2−*x*_0_ with *x*_0_ being the position of the mass center.

The hydrodynamic force and torque exerted by a fluid at *x_b_* are given by:

(18) $$F_{h}\left(x+\frac{\Delta t}{2}e_{i},\;t\right)=2e_{i}\left[f_{i}\left(x,t_{+}\right)-B_{i}\left(e_{i}\cdot u_{b}\right)\right],T_{h}\left(x+\frac{\Delta t}{2}e_{i},\;t\right)=x_{b}\times F_{h}$$

The properties of some lattice points would change when particles move, causing momentum exchange with the particles.

Aidun et al. [38] proposed a model of impact force and moment caused by the change of node properties. When the node changes from a fluid node to a solid one, the impact force and moment on the particles are:

(19) $$F_{c}\left(x,\;t\right)=\rho_{f}\left(x,\;t\right)u\left(x,\;t\right),T_{c}\left(x,\;t\right)=\left(x-x_{0}\right)\times F_{c}$$

where *ρ_f_* is the fluid density at the node.

Similarly, when the grid point in the solid particle at the previous time step becomes a fluid grid point in the current time distribution, the fluid at this grid point will also exert an impulse force and moment on the solid particle:

(20) $$F_{u}\left(x,\;t\right)=-\rho_{f}\left(x,\;t\right)u\left(x,\;t\right),T_{u}\left(x,\;t\right)=\left(x-x_{0}\right)\times F_{u}$$

Combining Equations (18)–(21), the total force and torque on the particle are given by Equations (21) and (22) during the time period [*t*, *t* +1]:

(21) $$F=\sum F_{h}\left(x+\frac{\Delta t}{2}e_{i},\;t\right)+\sum F_{c}\left(x,\;t\right)+\sum F_{u}\left(x,\;t\right)$$

(22) $$T=\sum T_{h}\left(x+\frac{\Delta t}{2}e_{i},\;t\right)+\sum T_{c}\left(x,\;t\right)+\sum T_{u}\left(x,\;t\right)$$

For a moving boundary, a stepped zigzag boundary for particle calculation is formed using the bounce scheme proposed and perfected by Ladd [37] and Aidun et al. [38]. This kind of method is the better way of treating boundaries when the mesh is fine enough.

Finally, the velocity and position of the particle are obtained through Newton’s second law.

### 2.3. Repulsive Force

The above method of calculating force or density reconstruction will fail if the distances between particles or between particles and walls are too small. Therefore, a short-range repulsion model should be introduced, i.e., the distances between particles or between particle and wall are not less than 1–2 lattice cells, thus the overlapping and collision of particles are also avoided.

The repulsive force is introduced [39]:

(23) $$f_{r}=\{\begin{array}{ll} \frac{C_{m}}{\varepsilon }{\left(\frac{d-d_{\min}-\Delta r}{\Delta r}\right)}^{2}e_{r},\; & d\;\leq d_{\min}+\Delta r\\ (0,0), & d\;>d_{\min}+\Delta r \end{array}$$

where *C_m_* =*MU*^2^/*a*, *M* is the particle mass, *U* is the velocity and *a* is the particle radius; *ε* = 10^−4^ is a positive coefficient; *d* is the distance between the centers of two particles or the distance between the center of particle and wall; ***e****_r_* is the direction vector; *d*_min_ = 2*a*; Δ*r* = 2Δ*x* represents two lattice cells when the repulsive force exists in the simulation.

### 2.4. Problem Definition

**Figure 1:**
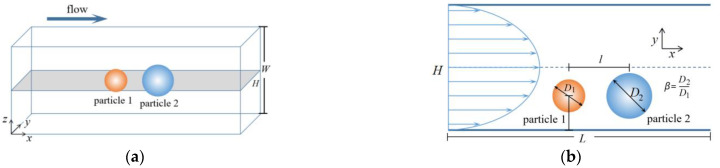
Two particles in Poiseuille flow of power-law fluid. (**a**) overall diagram, (**b**) on *x*-*y* plane.

Two rigid particles, initially on the same horizontal line, migrate in a Poiseuille flow of power-law fluid as shown in Figure 1. In a confined channel, when the Reynolds number is in a limited regime, the particles will move to the equilibrium position on the *x-y* plane under the combined action of inertial force, viscoelasticity, and lift induced by the walls (the existence of the wall creates a velocity gradient. One side of the particle near the wall has low velocity and high pressure, while the other side is opposite. The resulting pressure difference forms a lifting force). In the simulation, *L*/*H* = 14:1 (*L* = 2000Δ*x*) for balancing the computational efficiency and precision; *a* = 18 × lattice units; *H*/*W* ≤ 0.5 for ensuring that the number of equilibrium positions along the *y*-direction is only two sittings [22]; particle diameter *D_l_* = 18~45Δ*x* (*D_l_* is the diameter of a large particle); blockage ratio *k* = *D_l_/H* = 0.125~0.3, which reflects the influence of the walls on particle migration; the Reynolds number *Re* = *ρU*_max_^2−*n*^*H^n^*/*m* [40], where *ρ* and *U*_max_ are the fluid density and maximum velocity, *m* is the power-law consistency, the range of Reynolds numbers is 20 ≤ *Re* ≤60 because the inertial migration of particles has a certain limit on *Re* [41]; the diameter ratio of particle located downstream to that located upstream is *β* = 0.125~2.4; the power-law index of the fluid *n* = 0.4~1.2. A periodic constraint is enforced in the *x*-direction to replicate an infinite domain in the flow direction. In the computation, the calculated time steps are 12 × 10^6^ and 6 × 10^6^ when the particles move to *x/H* = 500 and *x/H* = 250, respectively, along the flow direction (each step is the time taken for particles to migrate one grid).

## 3. Validation

### 3.1. Velocity Profile for the Power-Law Fluid

For the Poiseuille flow of power-law fluid, there exist analytical solutions for the velocity given by Bird et al. [42]:

(24) $$u_{x}=U_{\max}\left[1-{\left(\left|1-\frac{2y}{H}\right|\right)}^{n+\frac{1}{n}}\right]\;for\;0\leq y\leq H,\;u_{y}=0$$

**Figure 2:**
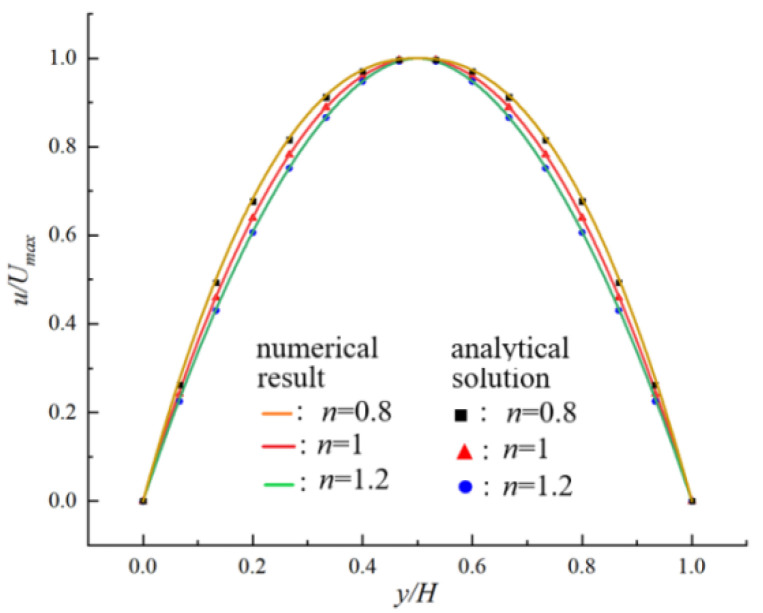
Comparison of numerical results.

The numerical results of fluid velocity are given in Figure 2 where the analytical solutions are given as a comparison. We can see that the numerical results are in good agreement with the analytical solution.

### 3.2. Particle Trajectories

**Figure 3:**
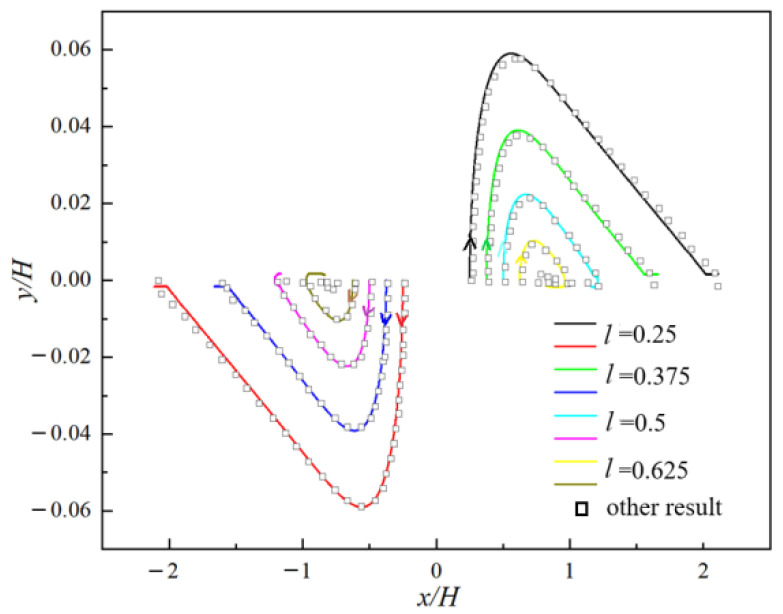
Comparison of trajectories of two analytical solutions of velocity profile, particles in a simple shear flow.

The trajectories of two particles in a simple shear flow are shown in Figure 3 where the other results [24] are also given as a comparison. In Figure 3, two particles with different initial horizontal distance *l* are placed on the center-line of the flow.

### 3.3. Grid and Compute-Domain Independence

**Figure 4:**
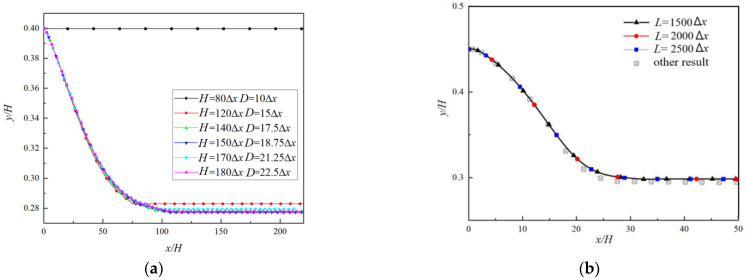
Comparison of results of particle trajectories for different *H* and *D* (**a**) and *L* (**b**).

The moving boundary is at the midpoint of the solid interior lattice and the fluid exterior lattice, which makes the boundary appear jagged and the calculation results oscillate, so a sufficient number of grids are required to ensure the stability of the calculation. To validate that the calculation results do not depend on the number of grids, the numerical results of particle trajectories for different *H* and *D* are shown in Figure 4a (the time to reach a steady state is 7200 s), based on which we select *D* = 18.75Δ*x* and *H* = 150Δ*x* in the following simulation for balancing the computational efficiency and precision.

In order to verify that the selected length of the flow does not affect the calculation results, the numerical results of particle trajectories for different length *L* are shown in Figure 4b (the time to reach a steady state is 2160 s) where other numerical results [27] are also given as comparison. It can be seen that there was only a little difference in the results for three *L*, so we select *L* = 2100Δ*x* (14*H*) in the following simulation.

## 4. Results and Discussion

### 4.1. Effect of Fluid Properties on the Steady State of Motion of Two Particles

#### 4.1.1. Two Particles of Same Diameter (*β* = 1)

**Figure 5:**
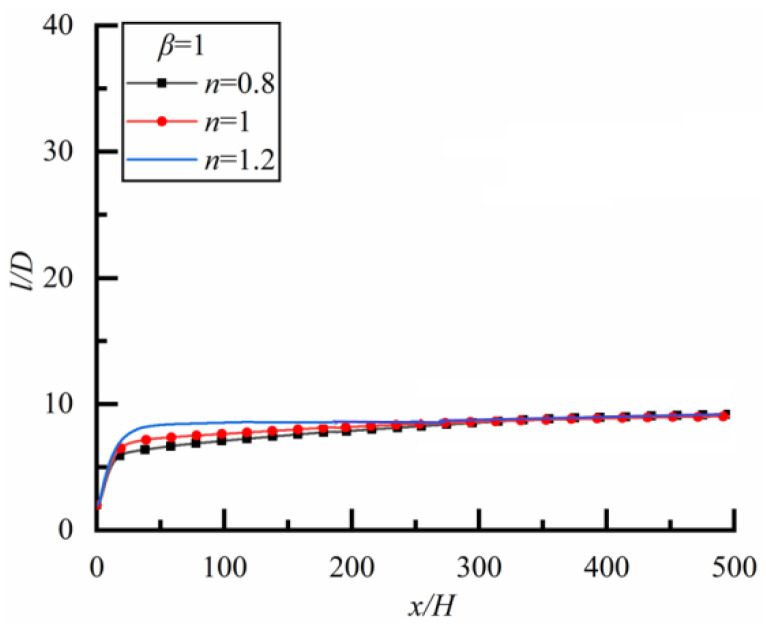
Changes of *l/D* along flow direction for different *n* (*Re* = 20).

Two particles of the same diameter (*β* = 1) are located initially on the same horizontal line with spacing *l* = 2*D* (*D* is the diameter of the large particle), the changes of the spacing of one of two particles along the flow direction for different power-law index n are shown in Figure 5 (the time to reach a steady-state is 28,800 s). We can see that *l*/*D* approaches 10 for three kinds of n, i.e., *l*/*D* will eventually stabilize at 10 regardless of whether it is in a Newtonian fluid, shear-thinning fluid, or shear-thickening fluid, the particle spacing in the steady-state is independent of fluid properties. In addition, it can be seen that the*l*/*D* increases rapidly at the initial stage (0 < *x*/*H* < 10), and then slowly.

#### 4.1.2. Two Particles with Different Sizes

**Figure 6:**
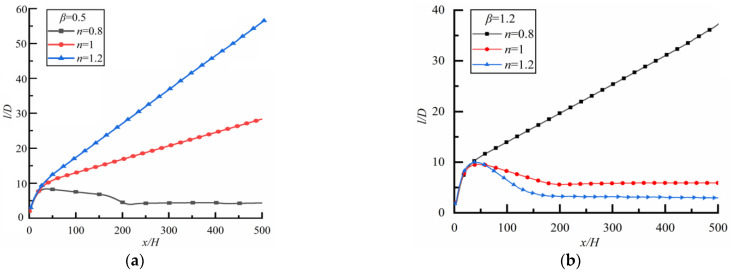
Changes of *l/D* along flow direction for different *n* (*Re* = 20). (**a**) *β* = 0.5, (**b**) *β* = 1.2.

Two particles with different sizes are located initially on the same horizontal line with spacing *l* = 2*D*. Figure 6 shows the changes of *l/D* along the flow direction for different *n*.

For the case of *β* = 0.5 (i.e., the small particle is downstream) as shown in Figure 6a (the time to reach a steady state is 18,000s), *l*/*D* increases rapidly at first, and then slowly and linearly in Newtonian fluid (*n* = 1).The case is the same as Newtonian fluid in shear-thickening fluid (*n* = 1.2), but the growth rate is greater for the latter. For shear-thinning fluid (*n* = 0.8), the case is completely different, *l*/*D* increases rapidly at first and then decreases slowly, finally approaching a constant, indicating that the particle spacing can reach a stable state finally.

For the case of *β* = 1.2 (i.e., the large particle is downstream) as shown in Figure 6b (the time to reach a steady-state is 21,600 s), *l*/*D* increases rapidly at first, then slowly and linearly in shear-thinning fluid, but *l*/*D* increases rapidly at first and then decreases in the shear-thickening fluid. In a Newtonian fluid, *l*/*D* increases rapidly at first and then decreases slowly, finally approaching a constant, and the particle spacing can reach a stable state finally. Therefore, the effect of fluid property on the particle spacing in the steady state is obvious.

### 4.2. Effect of Reynolds Number on the Steady State of Motion of TwoParticles

#### 4.2.1. Two Particles of the Same Diameter

##### Newtonian Fluid

**Figure 7:**
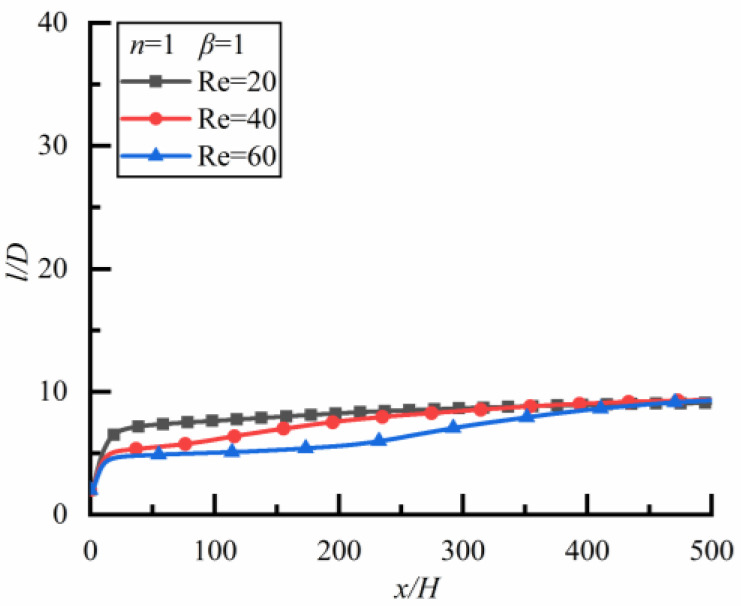
Changes of *l/D* along flow direction for different *Re* in a Newtonian fluid.

The changes of *l*/*D* along the flow direction for different *Re* are shown in Figure 7 (the time to reach a steady state is 32,400 s) where *l*/*D* approaches 10 for three kinds of *Re*, i.e., the particle spacing in the steady-state is independent of *Re* in a Newtonian fluid. In addition, the time for particles to reach a steady-state is the shortest for *Re* = 20, followed by *Re* = 40 and *Re* = 60, the smaller *Re* is, the shorter the time for the particles to reach the steady-state is.

##### Shear-Thinning Fluid and Shear-Thickening Fluid

**Figure 8:**
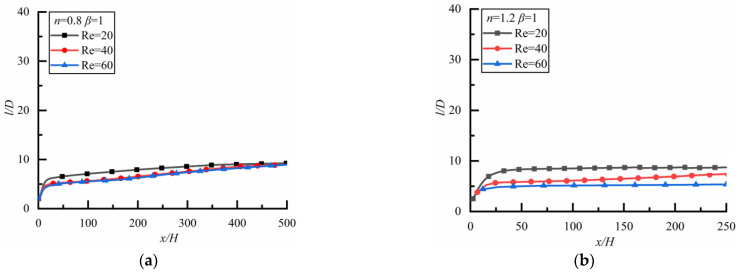
Changes of *l/D* along flow direction for different *Re*. (**a**) *n* = 0.8, (**b**) *n* = 1.2.

Figure 8 shows the changes of *l*/*D* along the flow direction for different *Re*. For the case of shear-thinning fluid, as shown in Figure 8a (the time to reach a steady-state is 33,120 s), *l*/*D* increases rapidly at first and then slowly, finally approaching ten for three kinds of *Re*, which is similar to the previous results [6,32,41]. However, the result is different in shear-thickening fluid as shown in Figure 8b (the time to reach a steady-state is 18,000 s). Although the particle spacing tends to be stable in the end, the values of *l*/*D* after stabilization are different. *l*/*D* is the largest for *Re* = 20, followed by *Re* = 40 and *Re* = 60.

#### 4.2.2. Two Particles with Different Sizes

##### Newtonian Fluid

**Figure 9:**
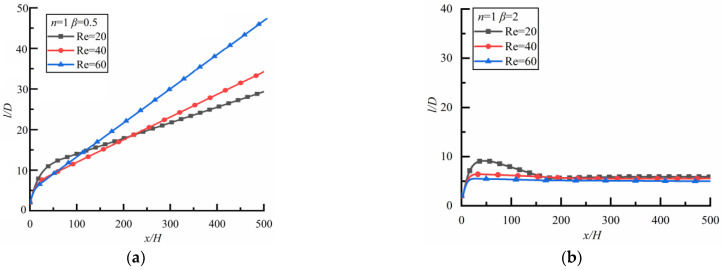
Changes of *l/D* along flow direction for different *Re* (*n* = 1). (**a**) *β* = 0.5, (**b**) *β* = 2.

Figure 9 shows the changes of *l*/*D* of two particles with different sizes along the flow direction for different *Re*. For the case of *β* = 0.5, as shown in Figure 9a (the time to reach a steady state is 7200 s), *l*/*D* increases rapidly at first and then slowly and linearly, but the growth rate is different for different *Re*. The larger *Re* is, the greater the growth rate is, and the larger the particle spacing is. The curves for *Re* = 60 *Re* = 40 are very close, so the change of *l*/*D* along the flow direction is not affected by *Re* at large *Re*. When a large particle is upstream, the wake area behind the large particle is relatively large, which directly affects the migration of small particle downstream, thus affecting the particle spacing. The characteristics of wake are related to *Re*, so there are different particle spacing at different *Re*.

For the case of *β* = 2, as shown in Figure 9b (the time to reach a steady state is 12,960 s), *l*/*D* finally approaches a constant for different *Re*. When a small particle is upstream, the wake area behind the small particle is relatively small and has little effect on the migration of a large particle downstream, thus *l*/*D* approaches approximately the same constant for different *Re*.

##### Shear-Thinning Fluid

**Figure 10:**
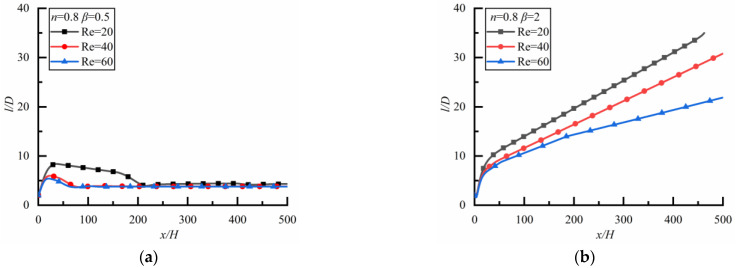
Changes of *l/D* along flow direction for different *Re* (*n* = 0.8). (**a**) *β* = 0.5, (**b**) *β* = 2.

The changes of *l*/*D* along the flow direction at different *Re* in the shear-thinning fluid are shown in Figure 10 where the result is opposite to that in a Newtonian fluid. For the case of *β* = 0.5 as shown in Figure 10a (the time to reach a steady state is 14,400 s), *l*/*D* approaches approximately the same constant for different *Re*. However, for the case of *β* = 2 as shown in Figure 10b (the time to reach a steady state is 18,000 s), *l*/*D* increases rapidly at first and then slowly, the larger *Re* is, the smaller the particle spacing is.

##### Shear-Thickening Fluid

**Figure 11:**
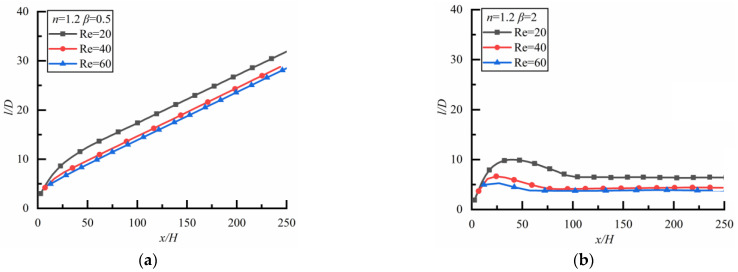
Changes of *l/D* along flow direction for different *Re* (*n* = 1.2). (**a**) *β* = 0.5, (**b**) *β* = 2.

Figure 11 shows the changes of *l*/*D* along the flow direction at different *Re* in the shear-thickening fluid. It can be seen that *l*/*D* increases rapidly at first and then slowly, and the particle spacing cannot reach a stable state when a large particle is upstream as shown in Figure 11a (the time to reach a steady state is 5040 s), which is similar to the case in a Newtonian fluid. On the contrary, the particle spacing will reach a stable state when a small particle is upstream as shown in Figure 11b (the time to reach a steady state is 8640 s).

From Figure 9b, Figure 10a, and Figure 11b, we can see that *l*/*D* approaches approximately the constant five for the case of two particles of different sizes, while approaching approximately the constant ten for the case of two particles of the same size as shown in Figure 5. This interesting phenomenon has not been reported before.

### 4.3. Stability Characteristics of Particle Spacing under Differentβand n

**Figure 12:**
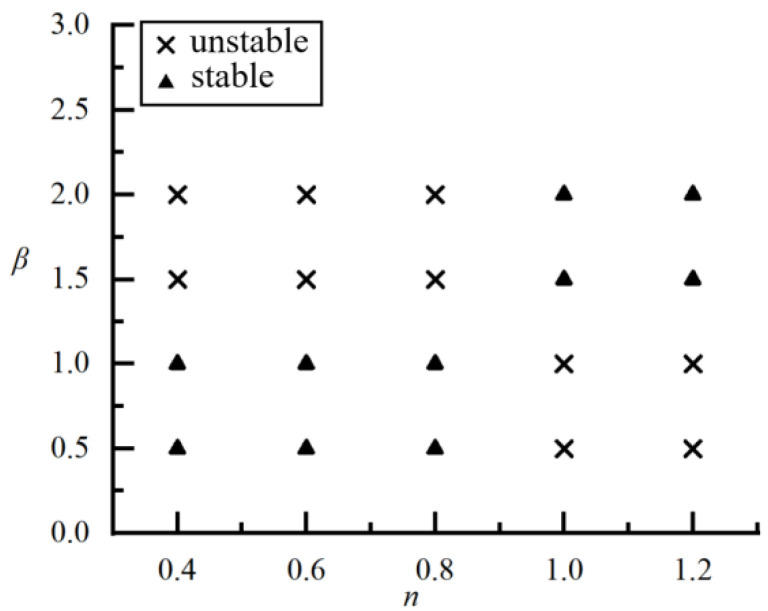
Phase diagram for the stability properties of particle spacing under different *β* and *n*.

In the simulation, it is found that the Reynolds number does not affect the stability characteristics of particle spacing within the range of *Re* in the present study but has a small effect on the value of particle spacing when a steady-state is reached. Therefore, only the stability characteristics of particle spacing at *Re* = 20 are analyzed here. The phase diagram for the stability properties of particle spacing under different *β* and *n* is shown in Figure 12 where we can intuitively see whether the final particle spacing is stable or unstable for a fixed *β* and *n*.

### 4.4. Effect of Non-Particle Spacing in Steady State

**Figure 13:**
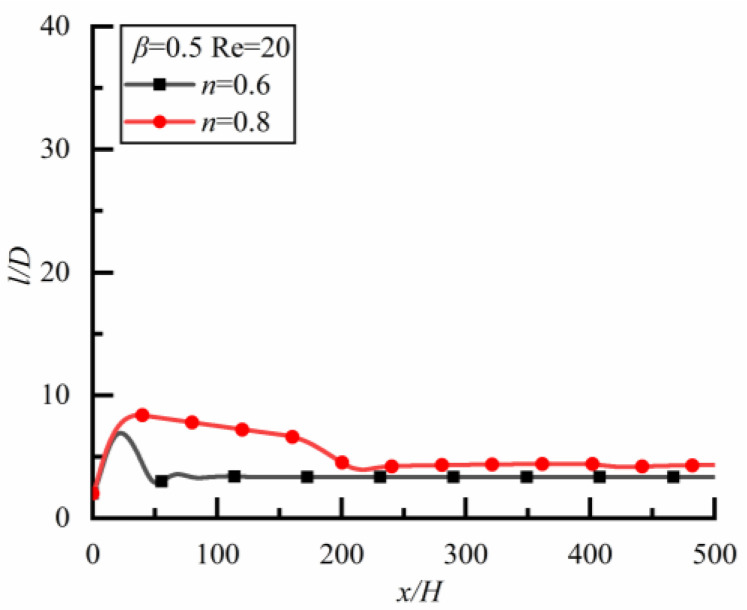
Relationship of particle spacing *l/D*.

**Figure 14:**
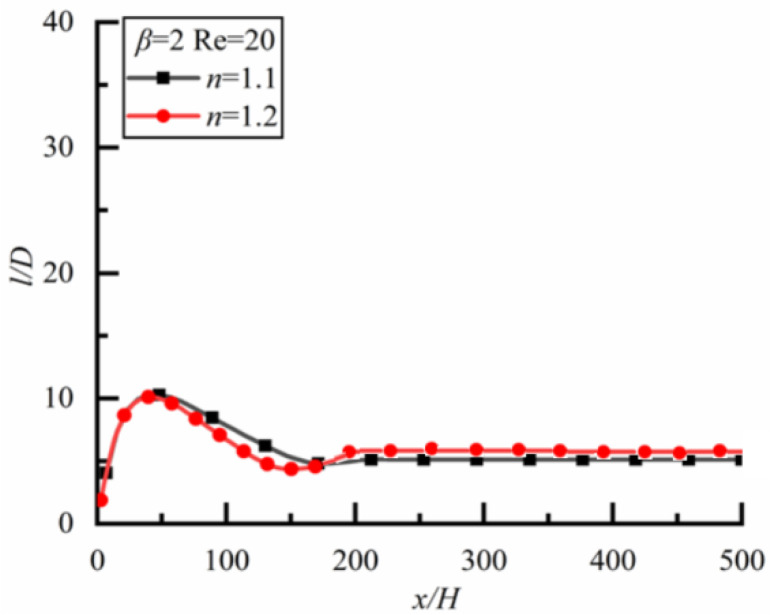
Relationship of particle spacing *l/D* and power-law index *n* (*Re* = 20, *β* = 0.5), and power-law index *n* (*Re* = 20, *β* = 2).

In Figure 10a, the particle spacing finally approaches a steady-state in shear-thinning fluid when the large particle is upstream. In order to illustrate the effect of power-law index *n* on the particle spacing, we give the relationship between *l*/*D* and *n* as shown in Figure 13 (the time to reach a steady-state is 18,000 s), it can be seen that the stronger the shear-thinning degree is, the smaller the particle spacing is. In Figure 11b, the particle spacing finally approaches a steady-state in shear-thickening fluid when the small particle is upstream, and the effect of power-law index non the particle spacing is shown in Figure 14 (the time to reach a steady-state is 15,120 s) where we can see that the degree of shear-thickening has little effect on the particle spacing in the steady-state.

### 4.5. Effect of βon Particle Spacing in SteadyState

**Figure 15:**
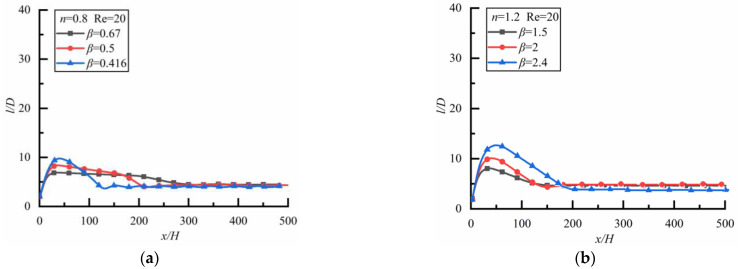
Relationship of particle spacing *l/D* and diameter ratio *β*(*Re* = 20). (**a**) *n* = 0.8, (**b**) *n* = 1.2.

The relationship between *l/D* and *β* is shown in Figure 15. When the large particle is upstream in shear-thinning fluid as shown in Figure 15a (the time to reach a steady-state is 21,600 s), the particle spacing in the steady-state approaches the same value for different *β*. The situation is the same when the small particle is upstream in shear-thickening fluid as shown in Figure 15b (the time to reach a steady-state is 20,160 s). Therefore, whether it is shear-thinning fluid or shear-thickening fluid, and whether a large particle is upstream or downstream, the particle spacing is the same when the spacing between the two particles finally tends to be stable.

## 5. Conclusions

The steady state of motion of two particles in the Poiseuille flow of power-law fluid is numerically studied using the LBM in the range of 20 ≤ *Re* ≤ 60, 0.125 ≤ *β* ≤ 2.4, and 0.4 ≤ *n* ≤ 1.2. Some results are validated by comparing the present results with available other results. The effects of *Re*, *β,* and *n* on the steady state of motion of two particles are discussed. The main conclusions are summarized as follows:

(1) For two particles of the same diameter, the particle spacing increases rapidly at the initial stage, and then slowly, and the particle spacing in the steady state is independent of the power-law index of fluid. In a Newtonian fluid, the particle spacing in the steady state is independent of *Re*. The smaller *Re* is, the shorter the time for particles to reach the steady state. In shear-thinning fluid, the particle spacing increases rapidly at first and then slowly, finally approaching 10 for different *Re*. In shear-thickening fluid, although the particle spacing tends to be stable in the end, the values of particle spacing after stabilization are different, the smaller *Re* is, the larger particle spacing is.
(2) For two particles of different sizes, the particle spacing does not always reach a stable state, and whether it reaches a stable state depends on the power-law index of the fluid. When the small particle is downstream, the particle spacing increases rapidly at first, then slowly and linearly in Newtonian fluid and shear-thickening fluid, but increases rapidly at first and then decreases slowly, finally approaching a constant in the shear-thinning fluid. In a Newtonian fluid, the particle spacing increases rapidly at first and then slowly and linearly, the larger *Re* is, the greater the growth rate is, and the larger the particle spacing is. In shear-thinning fluid, the particle spacing approaches approximately the same constant for different *Re*. The stronger the shear-thinning degree is, the smaller the particle spacing is. In shear-thickening fluid, particle spacing increases rapidly at first and then slowly, and cannot reach a stable state. The diameter ratio of two particles has no effect on the particle spacing in a steady state.

When the large particle is downstream, the particle spacing increases rapidly at first and then slowly and linearly in shear-thinning fluid, but increases rapidly at first and then decreases in the shear-thickening fluid. In a Newtonian fluid, the particle spacing increases rapidly at first, and then decreases slowly, finally approaching a constant. The effect of the power-law index on the particle spacing in the steady state is obvious. In a Newtonian fluid, the particle spacing finally approaches a constant for different *Re*. In a shear-thinning fluid, the particle spacing increases rapidly at first and then slowly; the larger *Re* is, the smaller the particle spacing is. In shear-thickening fluid, the particle spacing will reach a stable state.

## Data Availability

Not applicable.
